# Sustainable hydroponic production using solar energy and treated greywater within the water-energy-food-environment nexus

**DOI:** 10.1038/s41598-025-16030-4

**Published:** 2025-08-25

**Authors:** Mahmoud A. Abdelhamid, Sobhy M. Mahmoud, Mohamed K. Abou El-Nasr, Zhao Zhang, Zeinab M. Hendy

**Affiliations:** 1 Agricultural Engineering Department, Faculty of Agriculture, Ain Shams University, Cairo, 11241 Egypt; 2 Department of Horticulture, Faculty of Agriculture, Ain Shams University, Cairo, 11241 Egypt; 3 Key Laboratory of Agricultural Information Acquisition Technology, Ministry of Agriculture and Rural Affairs, China Agricultural University, Beijing, 100083 China

**Keywords:** Sustainability, Solar energy, Hydroponic, Treated greywater, Energy analysis, WEFE nexus, Energy science and technology, Engineering

## Abstract

Addressing the Water-Energy-Food-Environment (WEFE) nexus is critical for sustainable resource management. This study investigates a novel hydroponic system integrating photovoltaic (PV) solar energy and treated greywater (System-II), compared to a grid-powered system (System-I). Key performance indicators, including energy consumption, energy efficiency indices, and CO_2_ emissions, are evaluated for the two systems. Additionally, the morphological, physiological, and biochemical parameters of lettuce are measured. System-II achieved superior energy performance, with an energy ratio of 0.11, energy productivity of 0.16 kg/MJ, and specific energy of 6.14 MJ/kg, compared to System-I’s 0.05, 0.07 kg/MJ, and 14.89 MJ/kg, respectively. Additionally, the water use efficiency values were 0.071 kg/L for System-I and 0.073 kg/L for System-II. Moreover, System-II reduced CO_2_ emissions by over 94%, emitting only 0.0861 kg CO_2_ eq/m^2^, compared to 1.5386 kg CO_2_ eq/m^2^ from System-I. Morphological and physiological traits of lettuce irrigated with treated greywater remained optimal, showing a mean head weight of 682.9 g, head length and diameter of 17.7 cm and 18.3 cm, relative water content of 93.5%, 5.4% dry matter, and total chlorophyll content of 1.023 mg/g, comparable to those irrigated with tap water. This study highlights the potential of solar-powered hydroponics, utilizing treated greywater as a scalable and sustainable solution for efficient food production in alignment with WEFE nexus objectives. The findings provide insights into optimizing resource management in agricultural systems and contribute to the development of resilient, efficient, and sustainable food production systems in the face of global resource challenges.

## Introduction

The interdependence of Water, Energy, Food, and Environment (WEFE) resources has become a central focus in addressing global sustainability challenges. These sectors are intricately linked, where interventions in one area often have significant impacts on the others^1^. Agricultural activities significantly impact environmental sustainability through land use, greenhouse gas emissions, and water consumption^2,3^. Innovative solutions that promote resource efficiency are crucial to ensuring food security, energy sustainability, water efficiency, and environmental protection^4,5^. As population growth, urbanization, and climate change continue to intensify pressures on water, energy, food, and environmental systems, there is an urgent need for integrated and sustainable approaches to resource management. To meet the food requirements of a growing global population, global food production must increase by an estimated 60% by 2050. This intensifies pressure on natural resources, as agriculture currently accounts for approximately 70% of global freshwater withdrawals and nearly 30% of global energy consumption for food production and supply^6^. The pressures on water, energy, and food systems are further exacerbated by rapidly expanding populations and intensifying urbanization, placing additional stress on resource availability and ecosystem health ^7^.Moreover, the Commission on Sustainable Agriculture and Climate Change emphasizes the increasing correlation between population growth, rising food demand, and the need for sustainable intensification of food production^8^. Without integrated strategies that address the interconnectedness of water, energy, food, and the environment, efforts to achieve sustainability will remain fragmented and insufficient. Therefore, adopting a holistic WEFE Nexus approach is essential for ensuring the resilience and sustainability of these critical resource systems in the face of escalating global challenges.

Hydroponic farming is an advanced agricultural method that enhances water and space efficiency compared to conventional soil-based farming. It allows for precise control over nutrient and water delivery, reducing water wastage and increasing crop yields^9,10^. Despite these advantages, hydroponic systems require a continuous energy supply to operate pumps, nutrient delivery systems, and climate control mechanisms^11^. Therefore, ensuring the sustainability of these energy demands is critical for the long-term viability of hydroponic farming. On the other hand, renewable energy sources provide a sustainable alternative to conventional electricity derived from fossil fuels^12–15^. PV technology plays a critical role in enhancing the sustainability of hydroponic systems. While hydroponics is inherently efficient in water and nutrient use, it remains energy-intensive due to its reliance on continuous power for pumps, nutrient delivery, artificial lighting, and climate control systems. Integrating solar energy into hydroponic operations addresses this challenge by reducing dependence on non-renewable energy sources^16–18^. Therefore, solar-powered hydroponics has the potential to significantly lower the environmental footprint of controlled-environment agriculture while ensuring energy security in off-grid or resource-constrained areas^19^.

Greywater originates from kitchen and bathroom sinks, showers, and laundry, while blackwater contains urine, fecal matter, toilet paper, and toilet flushing water^20^. Reclaimed greywater can be used for non-drinking applications like irrigation, toilet flushing, landscaping, and replenishing aquifers, helping to mitigate water supply and demand imbalances in a region^21^. Treated greywater contains essential nutrients that can support plant growth, thereby reducing the need for synthetic fertilizers^22^. Additionally, greywater reuse addresses global water scarcity issues by repurposing water that would otherwise be discarded. However, ensuring the safety and effectiveness of greywater for hydroponic applications requires rigorous treatment and monitoring processes^23^. Moreover, the reuse of treated greywater hydroponic systems represents an innovative approach to conserving freshwater resources.

Despite the growing interest in sustainable agricultural practices, there is a notable knowledge gap in the comparative evaluation of hydroponic systems powered by PV solar energy versus those relying on conventional electricity, particularly when utilizing greywater as a primary water source. While hydroponics offers significant advantages in water and nutrient efficiency, the integration of renewable energy and unconventional water sources introduces complex dynamics that remain underexplored. A comprehensive understanding of the trade-offs and synergies between these systems is essential, especially in terms of performance, energy efficiency, crop yield, and environmental impacts^24^. This study directly addresses the WEFE nexus by promoting an integrated approach to resource management. It explores the efficient use of treated greywater (Water), the application of renewable PV solar energy for powering agricultural systems (Energy), the enhancement of crop production through hydroponic techniques (Food), and the potential for reducing carbon emissions and freshwater withdrawals, thereby mitigating environmental impacts (Environment). The primary objective of this study is to conduct a comparative analysis of the performance and efficiency of hydroponic systems powered by PV solar energy and conventional electricity sources, with a specific focus on the utilization of greywater treated as an irrigation resource. The study aims to evaluate key factors such as energy consumption, water use efficiency, crop productivity, and the associated CO_2_ emissions of these systems. The findings of this research contribute to the advancement of sustainable agriculture and resource-efficient management strategies, aligning with broader global sustainability goals. By demonstrating the feasibility and benefits of integrating renewable energy and greywater reuse in hydroponic food production, this study offers valuable insights to inform policy development and promote innovative practices that support the transition toward sustainable, resilient, and low-impact agricultural systems.

## Materials and methods

### Location and weather conditions

The proposed system was strategically installed on the rooftop of the Agricultural Engineering Department, Faculty of Agriculture, Ain Shams University, located in Shoubra El-Khima district, Qalyoubia Governorate (30° 5′ 10″ N latitude, 31° 12′ 44″ E longitude, and 23 m altitude), Egypt. This region is characterized by a semi-arid climate, making it a suitable testbed for sustainable irrigation technologies in water-scarce environments. This location choice brought several advantages, reinforcing its relevance to sustainability. Utilizing an educational building’s rooftop showcased innovative urban space use, transforming underutilized areas into productive agricultural sites. It also promoted awareness of renewable energy and sustainable practices among students and researchers, bridging the gap between theoretical knowledge and practical application. The system demonstrated the feasibility of integrating solar energy and greywater treatment techniques to address resource constraints, reduce carbon emissions, and improve urban agriculture. These findings are not only significant locally but also globally, providing transferable solutions for similar climates. This initiative highlights the pivotal role of educational institutions in advancing sustainability and resource-efficient agricultural practices.

**Fig. 1:**
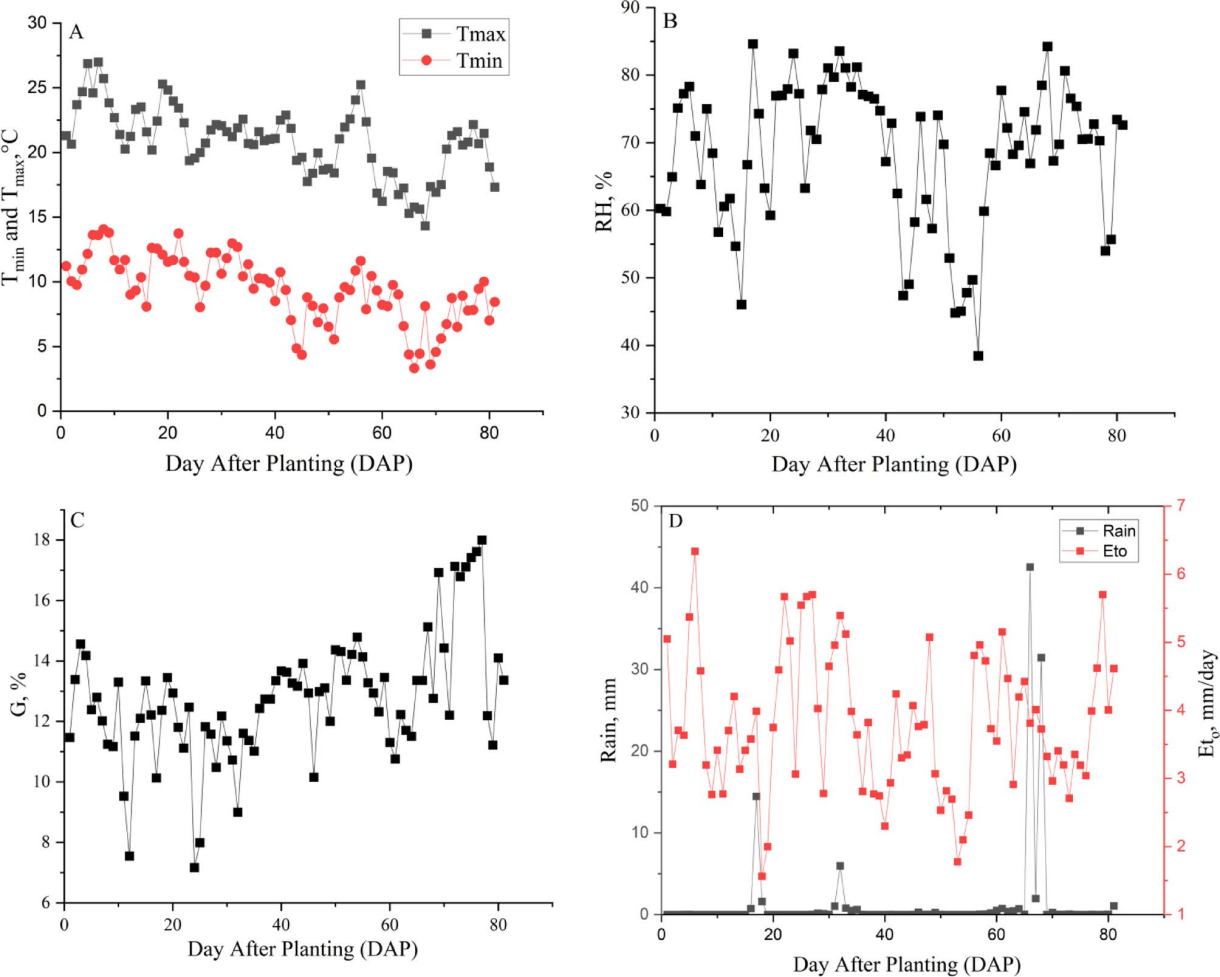
The meteorological parameters monitored during the 2023 growth season: (**A**) the temperatures, (**B**) the relative humidity, (**C**) the solar radiation, and (**D**) the rainfall rate and ET_o_.

The weather data monitored during the study highlighted critical variables influencing the system’s performance. The main weather variables were presented in Fig. 1, which includes the maximum (T_max_) and minimum air temperature (T_min_), average relative humidity (RH), solar radiation (G), and rainfall rate. The average air temperature was 15.1°C, while total precipitation was limited to 107.4 mm, with a daily average of 1.3 mm, emphasizing the need for efficient water management. Daily reference evapotranspiration (ET_o_) calculated using the FAO Penman–Monteith approach^25^, reached a maximum of 6.3 mm/day, with a cumulative value of 308.2 mm for the growing cycle. The average relative humidity was 68.3%, contributing to moderate ambient moisture, while solar radiation ranged from 7.2 MJ/m^2^/day to 18 MJ/m^2^/day, averaging 12.7 MJ/m^2^/day, ensuring ample energy for the solar-powered irrigation system. The average wind speed of 2.2 m/s provided moderate cooling and ventilation.

### Hydroponic farming setup

**Fig. 2:**
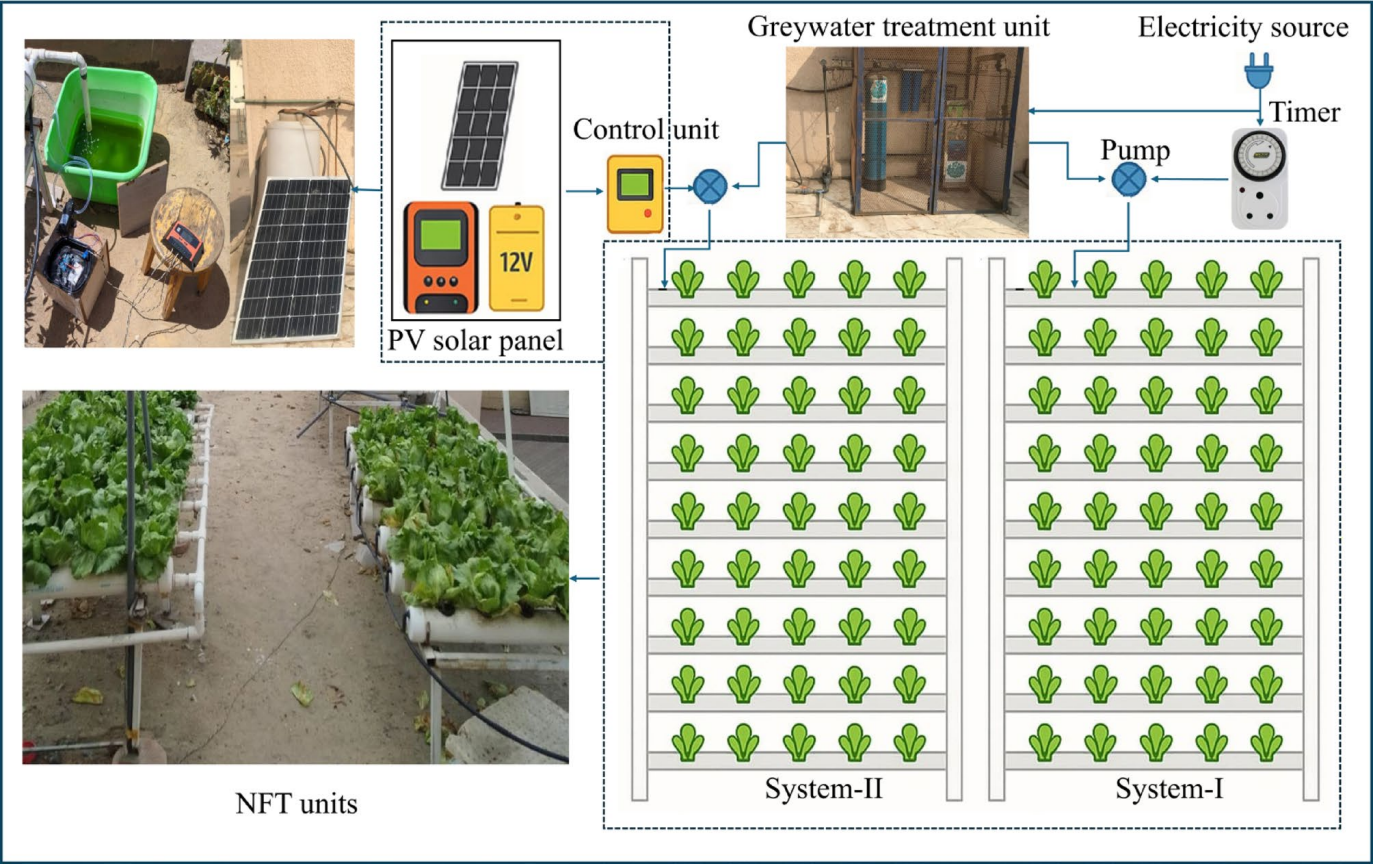
The layout of the hydroponic setup.

As illustrated in Fig. 2, the system consists of four key components: (i) a greywater treatment unit, responsible for supplying purified water to the hydroponic system, (ii) nutrient film technique (NFT) units, equipped with all necessary tools to ensure optimal growth conditions for the cultivated plants, (iii) a solar energy unit, which generates the power required to operate the NFT unit for System-II, and (iv) a water scheduling control unit, designed to regulate the timing and frequency of water pump within the NFT system, ensuring efficient water usage and plant health.

**Fig. 3:**
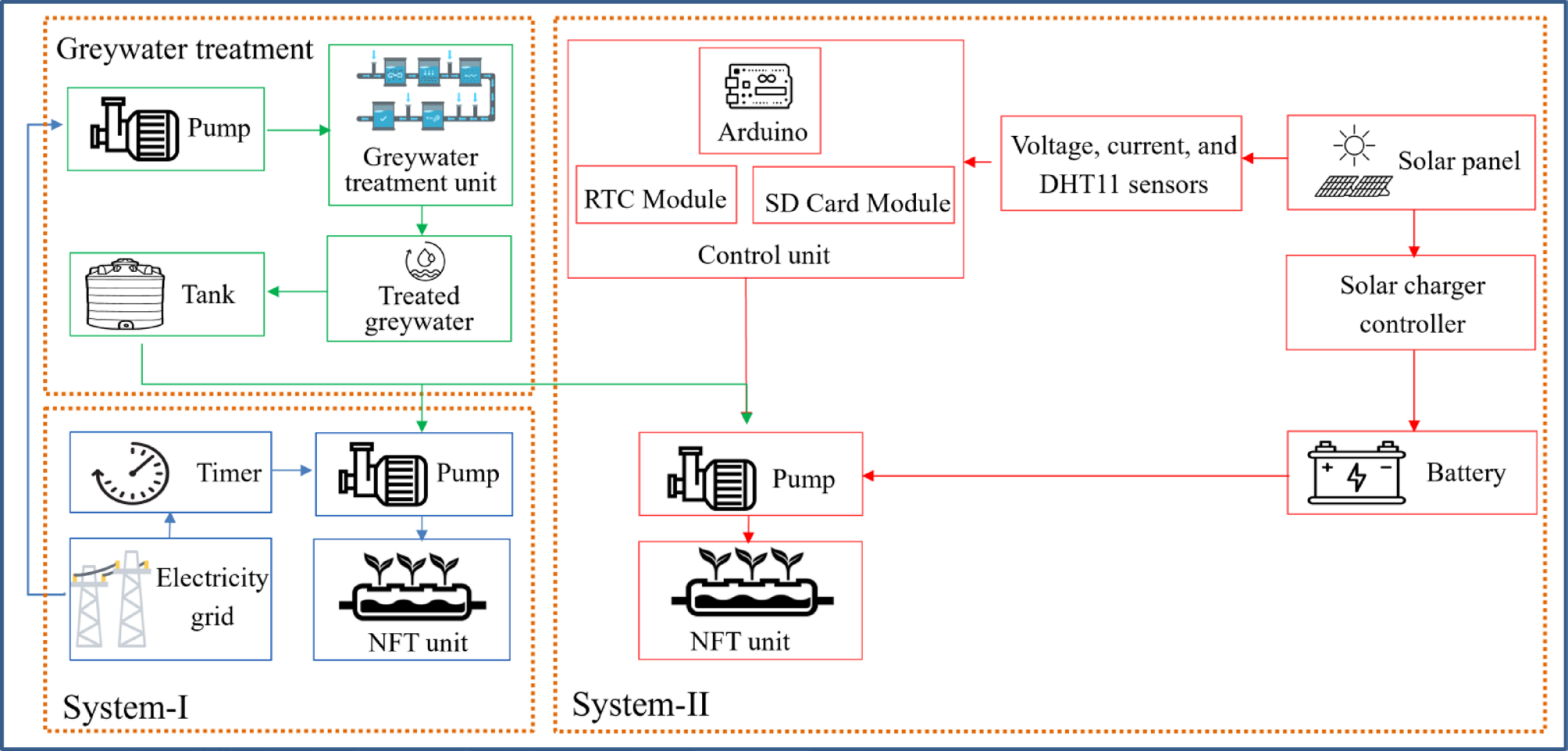
Methodology followed in the study.

The methodology of the sustainable hydroponic system is depicted in the flowchart Fig. 3. A sustainable and eco-friendly hydroponic system was developed to address water and energy challenges while ensuring efficient crop production. The system utilizes treated greywater for irrigation, reducing dependence on freshwater resources and promoting water conservation. The hydroponic pump in System-I and the treatment pump are powered by electricity from the grid, whereas in System-II, it is powered by solar energy, replacing conventional energy sources and minimizing environmental impact. The methodology integrates key components such as voltage and current sensors to monitor solar power availability. If the energy generated by the PV panel is sufficient, the pump is powered directly by solar energy. In the case of insufficient solar energy, a battery backup system is activated to maintain uninterrupted pump operation. The system’s performance and automation are managed by a microcontroller (Arduino Mega). The microcontroller receives input from sensors, including a DHT11 sensor to monitor temperature and humidity, an RTC module to manage time-based irrigation schedules, and an SD memory card module to log data for analysis. Greywater is pre-treated before entering the tank to ensure its suitability for irrigation, supporting a closed-loop, eco-friendly system. This innovative methodology ensures the sustainable use of natural resources, reduces pressure on freshwater supplies, and leverages renewable energy for agriculture, aligning with global sustainability goals.

#### Water treatment unit

The greywater supply for the treatment unit was sourced from the kitchen drainage of the educational building in the study area. The greywater treatment unit was installed on the rooftop, adjacent to the hydroponic system. Before any treatment, three greywater water samples were collected for analysis at the start, middle, and end of the experiment. Table 1 presents the physical and chemical analysis of the greywater and treated greywater. The treatment unit comprised a fiberglass-reinforced plastic (FRP) vessel with a capacity of 65 L and a working pressure of 10 bar, functioning as a sand filter. It also included two sediment filters equipped with Spun PP candles, offering filtration accuracy of 0.5 microns, housed in 20-inch bowls, and at the end, the treated greywater passed through a UV tube. A portion of the raw greywater was stored in a 100-L tank and transferred to the sand filter using a centrifugal pump with a power rating of 0.5 hp, a flow rate of 35 L per minute, and a pressure of 1 bar.

**Table 1:** Physical and chemical analysis for greywater and treated greywater.

Parameter	pH	TDS (mg/L)	BOD (mg/L)	COD (mg/L)	TSS (mg/L)	Turbidity (NTU)	Ammonia (mg/L)	Nitrate (mg/L)
Greywater	7.0	500	250	351.3	120.6	215	11.5	5.3
Treated greywater	6.7	300	15	33 .6	16.7	6.5	2.5	5.1

Sand filtration was employed as the primary filtration technique. This process utilizes sand as a medium to remove dirt particles as water percolates through its fine pores. A critical component of the sand filter was the multiport valve, located at its top, which allowed water to be directed in various pathways depending on the selected configuration. The multiport valve operated in two primary modes: filtration and backwash. During filtration, raw water entered the filter from the top, passed through the sand medium, and exited through the central pipe. Conversely, in the backwash process, water entered from the bottom via the central pipe, agitating the sand medium to dislodge dirt and debris, which was then expelled through the backwash outlet. The multiport valve had an inlet and outlet size of 1 inch and supported a maximum flow rate of 4000 L per hour.

Following sand filtration, the water underwent secondary treatment using sediment filters. These filters further eliminated residual particulate matter, including dirt and debris. The sediment filters, each equipped with a 10-inch filter housing and Spun PP candles with 0.5-micron accuracy, trapped pollutants on the outer surface of the candles while allowing clean water to flow through the center. The treated water was subsequently stored in a 100-L tank for use in irrigating the plants in the NFT units.

#### NFT units and plant growth

This study employed two identical NFT hydroponic units, System-I (grid-powered) and System-II (PV solar-powered), to evaluate the performance of sustainable lettuce cultivation under different energy regimes. Each unit consisted of nine parallel 1-m-long PVC pipes (4-inch diameter) mounted on a metal frame elevated 0.5 m above ground. Perforated holes (0.1 m diameter, spaced 0.2 m apart) held net pots containing lettuce plants initially propagated in perlite. A recirculating system delivered nutrient-rich treated greywater from a 60-L reservoir using a 100 W AC pump in System-I and an 80 W DC (4.5 L/min) pump in System-II. The nutrient solution, composed of 10 mL of Solution A and 20 mL of Solution B per liter of treated greywater, was maintained within a pH range of 6–7 and EC of 1.4–1.8 dS/m^26^. It was replaced weekly during the first two weeks and replenished every four days thereafter. This design ensured efficient nutrient delivery and minimal water waste, supporting forty plants per system and enabling a controlled comparison of energy and water management strategies.

#### PV solar panel and control unit

System-II is powered by a 100W monocrystalline solar panel, which supplies energy to a 12V lithium-ion battery through a solar charge controller. This control unit plays a critical role in managing and stabilizing the power supply to the hydroponic pump. The solar charge controller ensures optimal battery charging by regulating input from the solar panel, protecting the battery from overcharging and deep discharge. This function is essential for maintaining reliable operation, particularly under fluctuating weather conditions that affect solar irradiance as presented in Table 2. The hydroponic pump in the NFT system draws power directly from the battery, enabling a steady flow of nutrient-rich water to the crop roots.

**Table 2:** Components of System-II and their key functions.

Component	Function	Quantity
Arduino Mega	Serves as the central controller, receiving and gathering data from current and voltage sensors. It also stores the collected data onto an SD card for further analysis	1
ACS758 current sensor	Measures the output current generated by the PV panel	1
DC voltage sensor	Measures the output voltage generated by the PV panel	1
Solar charger controller	Regulates the current and voltage from the solar panel to the battery, preventing overcharging and ensuring battery health	1
PV panel (100W)	Generates electrical power by converting solar energy into direct current (DC) electricity	1
12V battery	Stores the electrical energy generated by the solar panel for use during non-sunny periods or when demand exceeds generation	1
DHT11 sensor	Measures environmental parameters, including PV panel temperature and humidity	1
RTC module (DS3231)	Provides precise real-time clock data, enabling accurate time-stamping of measurements and scheduled tasks	1
DC pump	Pumps water efficiently using DC electricity, ideal for solar-powered irrigation systems	1
Relay module	Regulated the run times of the watering pump based on the control signals from the Arduino	1
SD memory card module	Stored data from sensors, including voltage, current, and DHT11 sensor readings	1

**Fig. 4:**
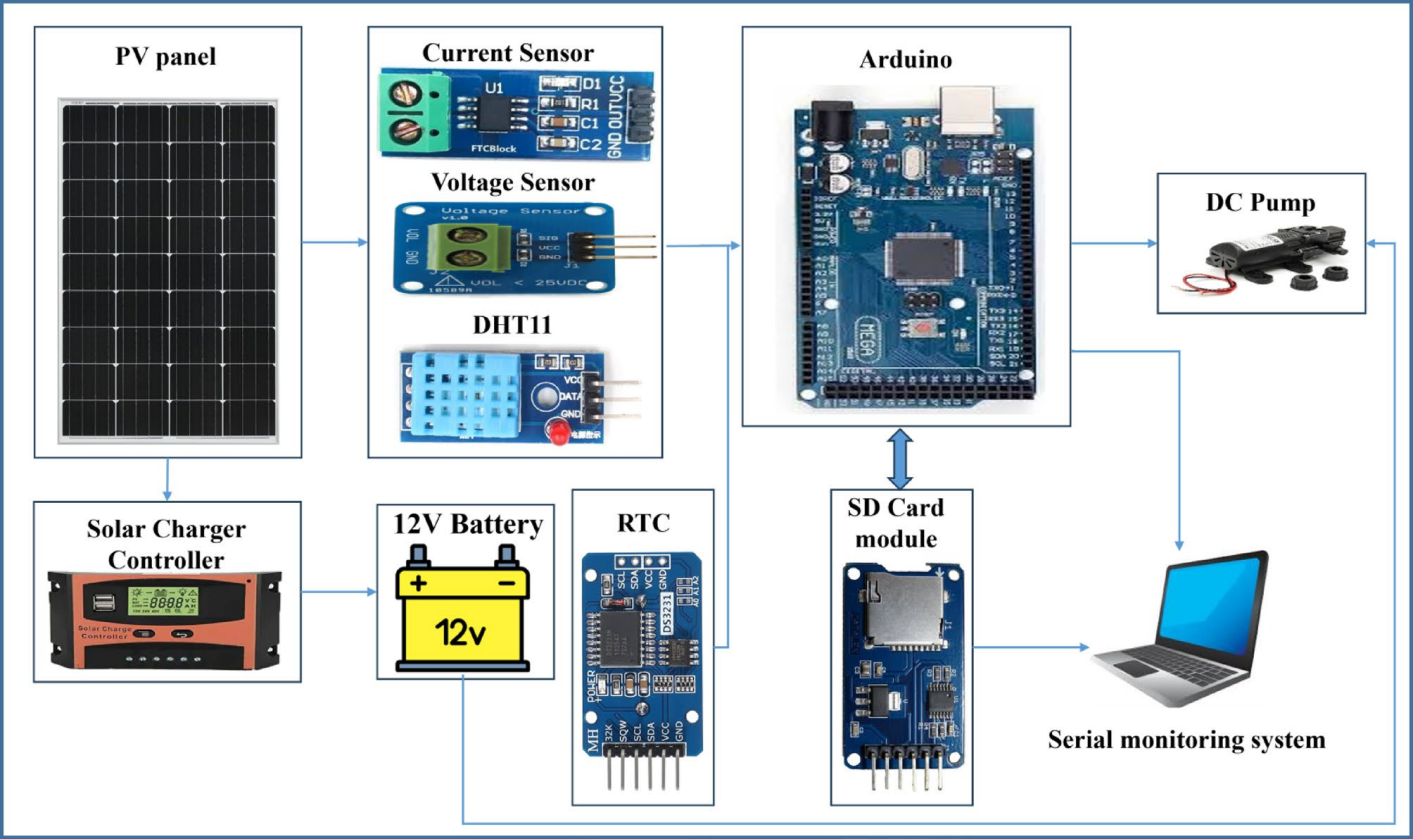
The circuit design of System-II.

A dedicated control system was designed using an Arduino Mega microcontroller to automate pump operation and environmental monitoring. The Arduino was programmed to manage the pumps’ on-and-off cycles based on predefined time intervals, thereby ensuring efficient water circulation while minimizing unnecessary energy consumption. It also interfaced with a DHT11 temperature-humidity sensor, a real-time clock (RTC) module, and an SD memory card module for continuous environmental data logging and system diagnostics. This integrated monitoring and control unit, illustrated in Fig. 4, enabled precise scheduling, efficient energy utilization, and improved system autonomy. The design effectively balanced sustainability and automation, optimizing water and energy usage in the solar-powered NFT hydroponic system.

The solar panel’s production was observed through monitoring the panel temperature and the current and voltage produced by the panel. Additionally, the operation of each NFT pump was scheduled to ensure efficient water use. The pump operating schedule was designed from 9:00 AM to 6:00 PM. It operates in intervals of 1:3, meaning it runs for 5 min (“life”) and then turns off for 15 min (“death”). The circuit design of System-II is illustrated in Fig. 4, while Table 2 presents a detailed breakdown of the components and their key functions.

### Measurements

#### PV solar panel performance evaluation

To assess the performance of the PV solar panel during the experimental period, ambient temperature and solar radiation were continuously monitored using high-precision instruments. Ambient temperature was measured using a K-type thermometer (model: DM6801A^+^, accuracy: ± 1.5 °C, China), ensuring accurate representation of environmental conditions. Solar radiation intensity was recorded with a solar meter (DBTU-1300, accuracy: ± 10 W/m^2^, USA), providing reliable data throughout the experiment. These measurements were essential for evaluating the panel’s performance under varying climatic conditions.

The output power *(P*_*output*_*)* of the PV panel was calculated as the product of measured voltage (V) and current (I). The electrical efficiency of the PV system (${\eta }_{el}$) was determined using Eq. (1), defined as the ratio of the electrical output power to the solar power input^27^:

1 $$\eta_{el} = \frac{{P_{output} }}{{P_{input} }} = \frac{V*I}{{A*G}}$$

where *A* is the surface area of the PV panel (m^2^), and *G* is the solar radiation (W/m^2^). This calculation enabled a comprehensive evaluation of the PV panel’s efficiency under real-time operating conditions.

#### Water consumption

At the onset of the experiment, a known-volume tank was used to monitor water consumption in the hydroponic systems. The tank was initially filled to its designated capacity. The volume of water lost from the tank was tracked to assess the water consumption of the systems. When the water level in the tank decreased below the predetermined threshold, water was added to restore it to the initial volume. The amount of water added during each replenishment was recorded. The intervals between water additions varied based on external factors such as weather conditions and the growth stage of the plants. The frequency and volume of water added were influenced by these variables, ensuring that the system met the water requirements for optimal plant growth throughout the growing season. At the time of harvest, the total amount of water added to the hydroponic systems over the entire cultivation period was calculated. This was achieved by summing up the volumes of water added during each recorded period of replenishment. The result represented the total water consumption of the hydroponic systems for the entire growing season.

#### Determination of energy consumption of the operating pump

The daily energy consumption of the pump was calculated using the following formula^27^:

2 $$E_{cp. daily} = \frac{P * t }{\eta }$$

The following equation calculates energy consumption during the growth period (gp)^27^:

3 $$E_{cp. gp} = E_{cp. daily} *d_{o}$$

where *P* is the pump power (W), *η* is the pump efficiency (0.8), *t* is the operating time of the pump in hours (h), and *d*_*o*_ is the number of days in the growth period.

#### Water use efficiency (WUE)

WUE is typically evaluated by measuring the weight of the harvested crop and dividing it by the total amount of water used for irrigation. In the context of this research, WUE (kg/L) represents the usable yield of lettuce produced per unit volume of water consumed. This efficient metric is critical for assessing the effectiveness of water utilization in agriculture. The calculation is performed using the following equation^27^:

4 $$WUE\left( {\frac{{{\text{kg}}}}{{\text{L}}}} \right) = \frac{{Lettuce\;yield~\left( {\frac{{{\text{kg}}}}{{{\text{m}}^{2} }}} \right)}}{{Water\;consumption~\left( {\frac{{\text{L}}}{{{\text{m}}^{2} }}} \right)}},$$

#### Energy use efficiency (EUE)

Energy use efficiency (EUE) is an important indicator that measures the efficiency with which energy consumption is converted into crop yield. It helps assess the effectiveness of energy use in crop production systems by quantifying the crop output per unit of energy consumed. In this study, EUE is calculated by evaluating the relationship between lettuce yield and energy consumption, as outlined in Eq. (4)^27^:

5 $$EUE\left( {\frac{{{\text{kg}}}}{{{\text{kWh}}}}} \right) = \frac{{Lettuce\;yield\left( {\frac{{{\text{kg}}}}{{{\text{m}}^{2} }}} \right)}}{{E_{{cp.gp}} \left( {\frac{{{\text{kWh}}}}{{{\text{m}}^{2} }}} \right)}}$$

#### Determination of morphological, physiological, and biochemical parameters

##### Determination of morphological parameters

Twenty plants were randomly selected to record agronomic traits. Plant height was determined biweekly by measuring the distance from the soil surface to the end of the longest leaf and leaf number. At harvest, shoot and root fresh weight parameters were measured, and dry matter was determined after drying in an oven at 70 °C^28^.

##### Determination of physiological parameters

For the chlorophyll (Chl) and carotene extraction^29^, one gram of fresh leaves was extracted in 10 mL of 80% acetone using a mortar and pestle. The filtered solution was determined by measuring the absorbance of the supernatant at 660, 640, and 440 nm for Chl a, Chl b, and carotene, respectively, by using a spectrophotometer (VWR, V-1200, accuracy: ± 0.5 nm, Palo Alto, CA, USA).

Total phenolic content was determined using the Folin-Ciocalteu reagent and measuring the absorbance at 765 nm using a spectrophotometer (VWR, V-1200, accuracy: ± 0.5 nm, Palo Alto, CA, USA. Using a gallic acid standard curve, the concentration of total phenolic compounds was determined and revealed as mg gallic acid equivalents (GAE)/100 g of DW^30^.

The relative water content (RWC) of leaves was measured according to Ahmed et al.^32^. The leaves were collected and then soaked in distilled water for a whole day at room temperature. The turgid weight of the leaves was measured after they were blotted dry using tissue paper. The leaves were placed in a hot air oven at 80 °C for 48 h, after which they were measured for dry weights. The relative water content was calculated as follows:

6 $$RWC \left( \% \right) = \frac{Fresh\;weight - Dry\;weight }{{Turgid\;weight - Dry\;weight}} \times 100$$

##### Determination of biochemical parameters

*Polyphenol oxidase activity (PPO)*

By Oktay et al.^33^, the rise in absorbance reading at 420 nm wavelength caused by the production of benzoquinone molecules was analyzed to determine the Polyphenol Oxidase enzyme activity (IU/mL enzyme) using a spectrophotometer (VWR, V-1200, accuracy: ± 0.5 nm, Palo Alto, CA, USA). In brief, 4 ml of extraction solution (0.1 M phosphate buffer, pH 7, 1% polyvinylpyrrolidone (PVP), and 0.1 mM EDTA) were mixed with one gram of leaf sample. The homogenates were centrifuged after 15 min of cooling. The supernatant was collected and used in enzymatic activity testing. The enzyme activity was measured by adding 0.6 mL of 0.1 M catechol to 2.3 mL of phosphate buffer (pH 6.5, 0.1 M) solution. Finally, the enzyme extract was added in 0.1 ml.

*Peroxidase activity (POD)*

Peroxidase activity was measured based on its capacity to change guaiacol into tetra guaiacol according to Polle et al.^34^. Briefly, one g of leaves was crushed in 4 mL of extraction phosphate buffer. 2.9 mL of the reaction mixture included the enzyme extract in addition to 10 mM H_2_O_2_, 20.1 mM guaiacol, and 100 mM phosphate buffer (pH 7.0). H_2_O_2_ was added, and the absorbance was measured for three minutes at 470 nm using a spectrophotometer (VWR, V-1200, accuracy: ± 0.5 nm, Palo Alto, CA, USA).

*MDA concentration*

The level of malondialdehyde (MDA) in the leaf tissue was determined by the thiobarbituric acid (TBA) by Dhindsa et al.^35^. Five milliliters of 1% (w/v) tri-chloroacetic acid (TCA) was used to homogenize 0.5 g of sample. For five minutes, the homogenate was centrifuged at 10,000 rpm and 4 °C using Rotina 380R (Refrigerated centrifuge Instruments, Hettich, Tuttlingen, Germany). Then, 1 mL of the supernatant was combined with 4 mL of 20% TCA containing 5% TBA. After 30 min of heating to 95 °C, the mixture was rapidly cooled in an ice bath. Following a 10-min centrifugation at 10,000 g, the value for the non-specific absorption at 600 nm was deducted from the absorbance of MDA at 532 nm. MDA’s extinction coefficient of 155 mM-1 cm-1 was used to determine its concentration according to Heath and Packer^36^.

*Leaf minerals content*

Immediately after the lettuce harvest, analysis of mineral content (N, P, K) was performed. Before measuring the elements, the samples were digested using the following method: 0.5 g of ground dry leaf powdered sample was digested in a digestion flask by boiling after adding 20 ml of concentrated H_2_SO_4_ until the mixture became clear. The digest was filtered into a 250 ml volumetric flask, and the solution was filled up to the mark with distilled water. Total nitrogen was determined by the Micro-Kjeldahl method according to Verma^37^ using Kjeldahl Analyzer (Velp UDK 129 Distillation Unit, Germany). Total phosphorus was determined calorimetrically by the ascorbic acid reduction method according to Watanabe and Olsen^38^; Total potassium was determined by using a flame photometer (model: 381, ± 2% up to 40 ppm, India) according to AOAC^39^.

### Evaluation of energy indicators

The energy consumption across various stages of the hydroponic farming life cycle was analyzed by quantifying each component and multiplying it by its corresponding energy equivalent coefficient, as presented in Table 3. To evaluate the system’s efficiency, the energy use efficiency, energy productivity, specific energy, and net energy gain were calculated using Eqs. (8–11)^40^:

7 $$Energy\;use\;Efficiency = \frac{{Energy\;output \left( {{\text{MJ}}\;{\text{m}}^{ - 2} } \right) }}{{Energy\;input \left( {{\text{MJ}}\;{\text{m}}^{ - 2} } \right)}}$$

8 $$Energy\;Productivity = \frac{{Lettuce\;yield \left( {{\text{Kg}}\;{\text{m}}^{ - 2} } \right)}}{{Energy\;input \left( {{\text{MJ}}\;{\text{m}}^{ - 2} } \right)}}$$

9 $$Specific\;Energy = \frac{{Energy\;input \left( {{\text{MJ}}\;{\text{m}}^{ - 2} } \right)}}{{Lettuce yield \left( {{\text{Kg}}\;{\text{m}}^{ - 2} } \right)}}$$

10 $$Net\;Energy = Energy\;output \left( {{\text{MJ}}\;{\text{m}}^{ - 2} } \right) - Energy\;input \left( {{\text{MJ}}\;{\text{m}}^{ - 2} } \right)$$

**Table 3:** System elements of energy equivalents in hydroponic farming.

Input and output	Energy equivalent (MJ/unit)	References
Input		
Human labor (hr)	1.96	^41,42^
Stationary equipment (kg yr)	9	^41^
Electricity (kWh)	11.93	^41^
Seeds	14.7	^41^
Pesticides (kg)	199	^41^
Phosphate (P_2_O_5_)	12.44	^43^
Potassium (K_2_O)	11.15	^43^
Nitrogen (N)	66.14	^41,43^
Output		
Lettuce (kg)	0.7	^44^

### CO_2_ emissions evaluation

The assessment of system CO_2_ emissions serves as a crucial metric to determine whether the proposed hydroponic system contributes to net CO_2_ emissions or achieves a net reduction by leveraging solar energy for operation. Total CO_2_ emissions were computed by assessing each input used in the hydroponic system for 1 m^2^ of System-I and System-II and multiplying it by the corresponding CO_2_ equivalent coefficient, as detailed in Table 4^41^.

**Table 4:** System elements of CO_2_ equivalents in hydroponic farming.

Input	CO_2_ emissions equivalent	Unit
Input		
Electricity	0.210	Kg CO_2eq_ kWh^−1^
Steel	1.76	kg CO_2eq_ m^−2^
PE	2.2	kg CO_2eq_ m^−2^
PVC	3	kg CO_2eq_ m^−2^
Phosphate	0.2	kg CO_2eq_ kg^−1^ (P_2_O_5_)
Potassium	0.15	kg CO_2eq_ kg^−1^ (K_2_O)
Nitrogen	1.3	kg CO_2eq_ kg^−1^ (N)

## Results and discussion

### PV solar panel performance analysis

**Fig. 5:**
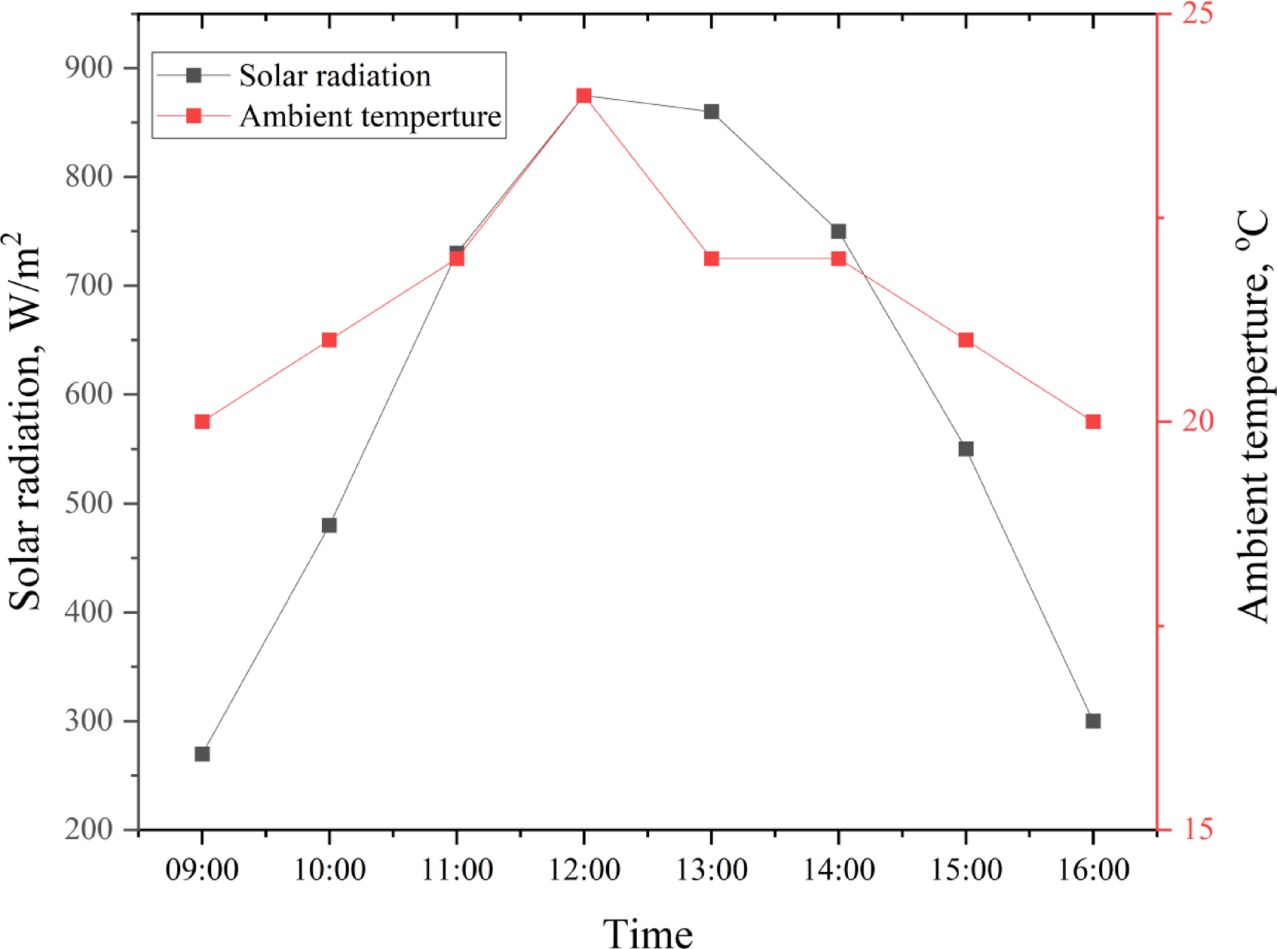
The solar radiation and ambient temperature of an experimental day in February 2023.

The PV solar panel was evaluated during an experimental day in February 2023. The solar radiation and ambient temperature were measured and analyzed over a day, as shown in Fig. 5. Solar radiation exhibited a sharp increase from 9:00 AM, peaking at approximately 875 W/m^2^ at 12:00 PM, before gradually declining to around 300 W/m^2^ by 4:00 PM. This trend reflects the natural diurnal variation in solar intensity, driven by the sun’s position. Similarly, the ambient temperature rose from approximately 20°C at 9:00 AM to a maximum of 24°C at 12:00 PM. The observed positive correlation between solar radiation and ambient temperature highlights the influence of solar intensity on environmental thermal conditions. These findings have significant implications for hydroponic systems powered by PV solar energy. The peak solar radiation period, occurring between 10:00 AM and 2:00 PM, which matches Elsaid et al.^45^, represents the optimal window for energy generation, ensuring sufficient power availability for system operations.

**Fig. 6:**
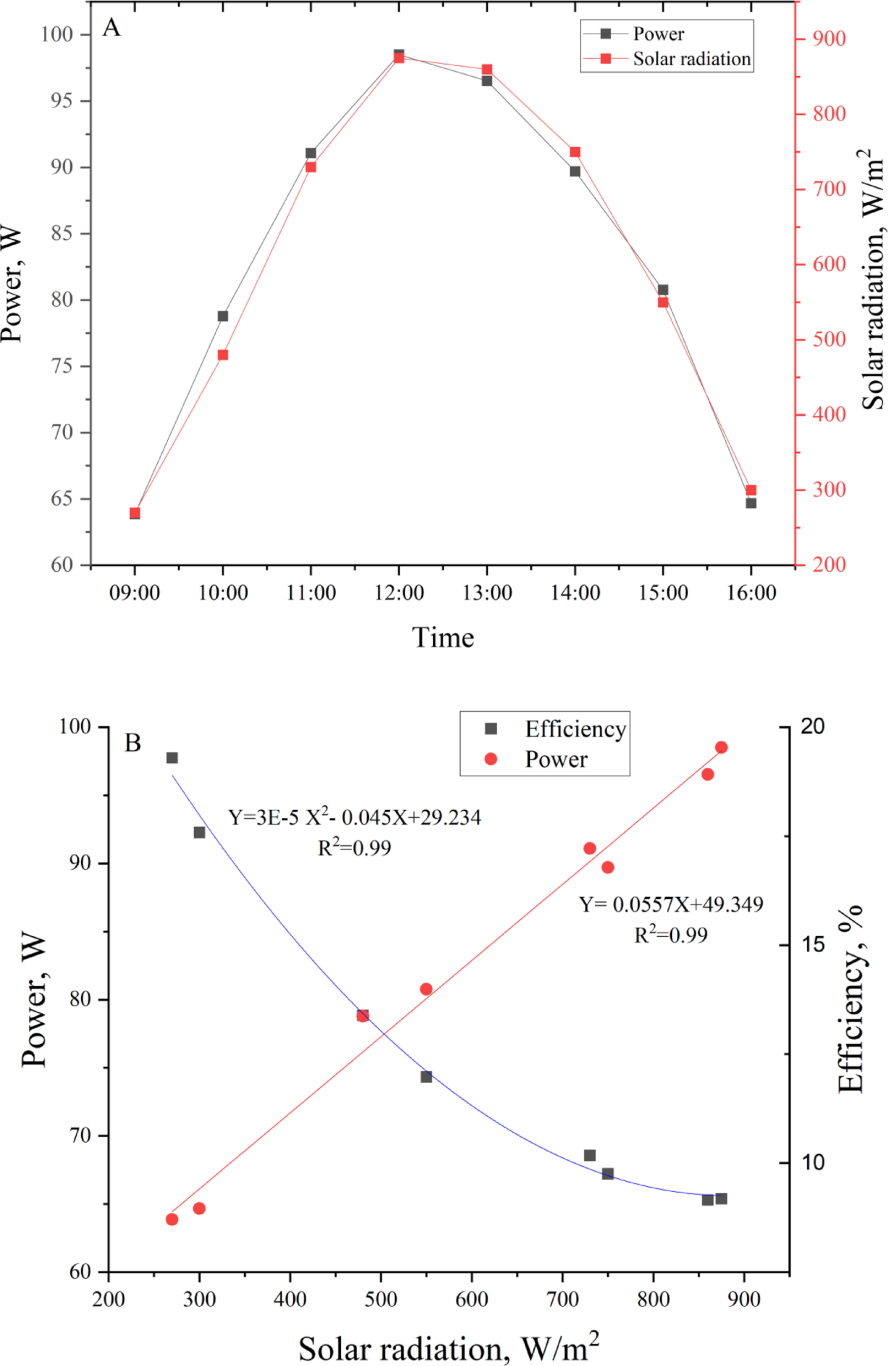
**S**olar power output and solar radiation vs time (**A**) and relationship between solar radiation with PV solar panel power and efficiency (**B**).

Figure 6 provides insights into the relationship between solar radiation and power output over time and their direct correlation. In Fig. 6A, the time series data illustrates how power output (in watts) and solar radiation (in W/m^2^) vary throughout the day, plotted on dual y-axes. Both variables exhibit a similar trend, starting at a low value in the morning (around 9:00), steadily increasing to a peak at approximately 12:00 PM, and then gradually declining toward 16:00 PM. This pattern reflects the natural diurnal variation in solar radiation as the sun rises, reaches its zenith, and sets. The close alignment of the curves indicates a strong dependency of power generation on the availability of solar radiation. The peak power and radiation levels suggest maximum system output during midday, aligning with the period of maximum solar irradiance. Figure 6B further explores the relationship between solar radiation and power output by plotting power output as a function of solar radiation. The data points are fitted with a linear regression model. The high R^2^ value of 0.99 suggests an excellent fit, indicating that the model effectively captures the relationship between these variables. The curve demonstrates a linear trend, with power output increasing as solar radiation rises. Overall, this information can aid in optimizing solar energy systems by identifying the critical factors affecting performance under varying radiation levels.

Figure 6B depicts the relationship between solar radiation (W/m^2^) and PV solar panel efficiency (%) using a quadratic regression model. As solar radiation increases, efficiency exhibits a clear downward trend, with the highest efficiencies observed at lower radiation levels (close to 20% around 270 W/m^2^). In contrast, at higher radiation levels (above 800 W/m^2^), efficiency drops to values below 10%. This inverse relationship is modeled by the quadratic equation Y = 3E−5X^2^−0.045X + 29.234, where Y is efficiency and X is solar radiation. The high coefficient of determination (R^2^ = 0.99) confirms that the quadratic model accurately captures this behavior. The observed decline in efficiency with increasing solar radiation can be attributed to several factors. One significant reason is the thermal effects on photovoltaic systems. Higher solar radiation often leads to increased temperatures of solar panels, which reduces their efficiency due to greater resistive losses in the system. Additionally, at elevated radiation levels, the system may approach its operational saturation point, where the incremental gain in power output does not match the increase in energy input.

### Analysis of WUE, EUE, and energy consumption for System-I and System-II

The analysis of seasonal resource use and efficiency metrics between System-I and System-II provides insights into their performance and sustainability. Both systems produce roughly the same yield with an average of 11.38 kg/m^2^ of lettuce per season and use roughly the same water, but energy consumption is different. System-I consumes 161 L of water per m^2^, which is slightly more than the System-II, which uses 155 L per m^2^. This difference is reflected in the WUE, with System-II achieving a slightly higher value (0.073 kg/L) than System-I (0.071 kg/L), indicating that there is no noticeable difference in water use between the two systems. According to Steduto et al.^46^ WUE is a critical metric in sustainable agriculture, as higher efficiency implies better resource management. However, given the minimal difference observed, it can be concluded that both systems are effectively utilizing water resources with negligible disparity.

**Fig. 7:**
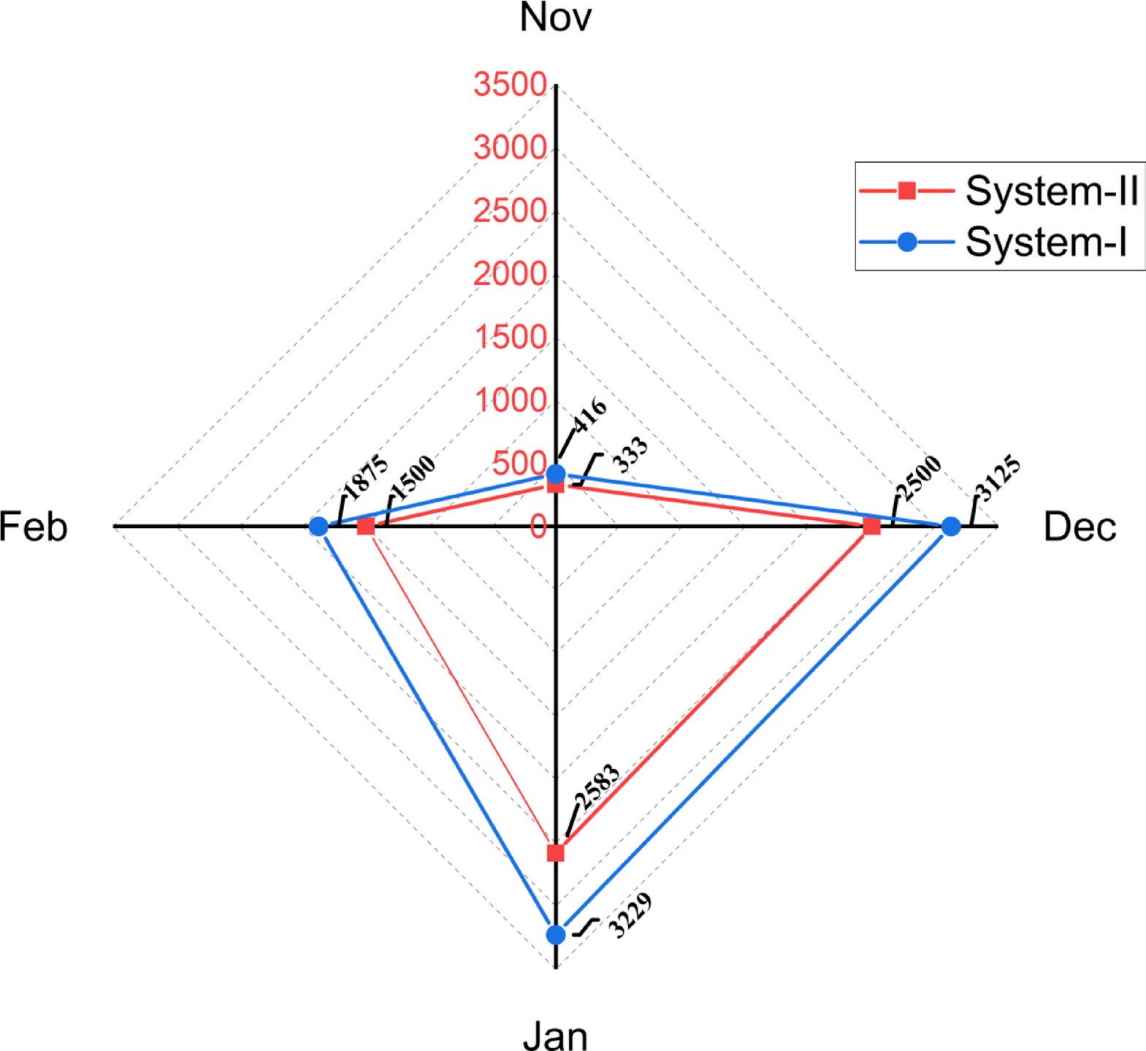
Energy consumption during growth stages for System-I and System-II.

Figure 7 illustrates the performance of System-I and System-II over four months (November to February) based on recorded energy consumption values for 1 m^2^. System-I consistently demonstrated higher energy consumption values in December (3125) and January (3229), whereas System-II exhibited slightly lower values during the same months (2500 in December and 2583 in January). Both systems recorded minimal energy consumption during November, with values converging at 333 for System-II and 416 for System-I, representing the lowest energy consumption across the study period. In terms of energy consumption, System-II uses 6.9 kWh/m^2^ per season, while System-I requires 8.6 kWh/m^2^. System-II demonstrates a higher EUE at 1.646 kg/kWh, compared to System-I’s 1.316 kg/kWh. These findings align with previous studies indicating that optimized energy use enhances productivity in controlled environment agriculture^47^. Higher EUE in System-II suggests that it is more energy-efficient, reducing operational costs and environmental impact. These results emphasize the need to use PV due to the sustainability benefits they offer through reduced greenhouse gas emissions and alignment with the WEFE nexus principles. The WEFE nexus emphasizes the interconnection between these critical resources, advocating for integrated solutions^2–4^. Implementing renewable energy sources such as PV can mitigate the high energy demand observed in System-I, aligning with sustainable agricultural practices.

## Morphological, physiological, and biochemical analysis

The physical traits of lettuce plants were evaluated based on head weight, head length, and head diameter. It is found that lettuce plants irrigated with the treated greywater had a mean head weight of around 682.9 g, and the length and diameter of the head were 17.7 and 18.3 cm, respectively, as can be seen in Table 5. Besides, lettuce plants had reached the RWC of 93.5%, with 5.4% of dry matter. These results are relative to those reported by Kaney^48,49^. Through the results obtained and comparing them with the average values of these characteristics under irrigation with tap water, no differences were observed between them and the treated greywater.

**Table 5:** Impact of greywater treatment on morphological, physiological, and biochemical traits of crisphead lettuce under hydroponic system.

Category	Parameter	Value (Mean ± SE)
Morphological Parameters	Head weight (g)	682.9 ± 9.634
	Head height (cm)	17.7 ± 0.127
	Head diameter (cm)	18.3 ± 0.267
	Dry matter (%)	5.4 ± 0.220
Physiological Parameters	Chlorophyll a (Chl a)	0.790 ± 0.024
	Chlorophyll b (Chl b)	0.232 ± 0.007
	Total chlorophyll	1.023 ± 0.014
	Carotene	0.220 ± 0.007
	Total phenolic content	2.805 ± 0.323
	Relative water content (RWC)	93.5 ± 0.089
Biochemical Parameters	Polyphenol oxidase (PPO, EU/ml)	0.694 ± 0.016
	Superoxide dismutase (SOD, EU/ml)	37.823 ± 0.181
	Malondialdehyde (MDA, nmol/g)	191.339 ± 0.002
	Nitrogen (N, %)	3.044 ± 0.082
	Phosphorus (P, %)	0.285 ± 0.007
	Potassium (K, %)	2.557 ± 0.040

As shown in Table 5, the range of total chlorophyll found in the current study (1.023 mg/g) was consistent with Ahmed^28^ and relatively lower than Phibunwatthanawong and Riddech^50^, who reported a range between 1.8 and 2.8 mg/g, and higher than Fallovo et al.^51^, who observed values between 0.521 and 0.793 mg/g in hydroponically grown lettuce.

The biosynthesis of phenolic compounds is mainly mediated by two enzymes, phenylalanine ammonium lyase and tyrosine ammonium lyase, which participate in the pathway of shikimic acid. The total phenolic content of treated greywater irrigated plants (2.805 g GAE/100 g DW), as shown in Table 5 was consistent with Ahmed^28^, who found the value between 2.7 and 3.1 g GAE/100 g DW. Based on these findings, the treated greywater irrigated hadn’t had an impact on total phenolic content in lettuce leaves, and with the low value, there is no effect induced by plant stress. However, such observations need further investigation, examining relevant plant stress indicators.

Data tabulated in Table 5 showed that antioxidant enzyme PPO and SOD achieved value 0.694 and 37.823 EU/ml, respectively. Our findings are in parallel with those of Kučerová et al.^52^ who reported that control treatment gave a value around 40 U/mg, which indicates that the treated water did not increase the oxidative stress in plant cells compared with plants irrigated with tap water. The value of MDA was detected in plants grown in hydroponic recording 191.339 nmol g^−1^; this result was lower than Abu-Shahba et al.^53^ who recording value is around 500 nmol g^−1^, which indicates that there is no oxidative stress where it is MDA is an indicator of oxidative damage from lipid peroxidation by ROS of polyunsaturated fatty acids^54,55^.

The data presented in Table 5 revealed the effect of greywater on mineral content; the results indicated that the percentage of nitrogen, phosphorus, and potassium were 3.044, 0.285, and 2.557%, respectively. These results are consistent with Kaney^49^.

### Analysis of energy input and output for two systems

The comparison of energy input and output between the two hydroponic systems, System-I and System-II, reveals significant differences in energy efficiency and sustainability. As illustrated in Table 6, both systems consumed identical energy for labor, nutrients, seeds, electricity for the treatment pump unit, and stationary equipment. These values demonstrate that the baseline requirements for nutrient supplementation, labor, and equipment remain consistent, irrespective of the energy source. However, a major distinction lies in the energy required for the NFT pump. System-I consumes 99.6 MJ/m^2^ of energy for the pump, whereas System-II entirely offsets this with PV solar energy, consuming no conventional electricity for the same operation. Both systems yield the same lettuce energy output of 7.9 MJ/m^2^. The integration of PV solar energy in System-II significantly enhances its sustainability by reducing reliance on non-renewable energy sources, thereby lowering overall carbon emissions. Previous studies have demonstrated that PV-powered agricultural systems can lead to substantial reductions in greenhouse gas emissions and long-term energy cost savings^27^. Moreover, transitioning to renewable energy aligns with global sustainability goals, promoting environmentally friendly agricultural practices. The findings from this study reinforce the potential of PV solar energy to contribute to sustainable hydroponic production by enhancing energy efficiency without altering other essential input requirements, such as labor and nutrient supplementation.

**Table 6:** Input and output energy for producing 1 m^2^ of lettuce in System-I and System-II.

Input	Quantity per m^2^		Energy (MJ/m^2^)	
	System-I	System-II	System-I	System-II
Labor	0.2	0.2	0.392	0.392
Electricity for the treatment pump unit	0.037	0.037	0.44	0.44
Nitrogen	0.014	0.014	0.91	0.91
Phosphorous	0.012	0.012	0.14	0.14
Potassium	0.046	0.046	0.50	0.50
Seeds	0.0002	0.0002	0.003	0.003
Electricity/ PV for NFT pump	6.917	0	99.6	0
Stationary equipment	7.5	7.5	67.5	67.5
Output			–	–
Lettuce (kg)	11.38	11.38	7.9	7.9

The substitution of conventional electricity with PV solar energy in System-II significantly enhances energy efficiency and sustainability. By eliminating dependence on non-renewable electricity for the NFT pump, System-II reduces overall energy demand and minimizes greenhouse gas emissions. Additionally, System-II aligns with renewable energy goals, demonstrating its potential for reducing operational costs over time. Despite these benefits, the nutrient and labor inputs remain unaffected by the energy source, indicating that renewable energy integration primarily influences the energy efficiency of the system without altering other input requirements. This comparative analysis underscores the environmental and economic advantages of utilizing PV solar energy in hydroponic systems, highlighting its potential to create sustainable agricultural solutions while addressing the WEFE nexus.

The analysis of energy indices for System-I and System-II is illustrated in Table 7. The ER is significantly higher in System-II (0.11) compared to System-I (0.05). This indicates that System-II is more energy-efficient, producing a greater output per unit of energy input. Similarly, EP is more favorable for System-II (0.16 kg/MJ) than System-I (0.07 kg/MJ), highlighting the enhanced productivity of the PV-powered system. These findings align with previous studies that emphasize the role of renewable energy in improving agricultural energy efficiency and reducing environmental impact^56^. The SE is substantially lower in System-II (6.14 MJ/kg) compared to System-I (14.89 MJ/kg). This finding reflects the reduced energy demand of System-II, largely attributed to its reliance on renewable solar energy rather than conventional electricity. Finally, the NEG is less negative in System-II (− 61.93 MJ) than in System-I (-161.53 MJ). While both systems operate with an energy deficit, the improved NEG of System-II indicates a more sustainable energy balance. These findings emphasize that System-II outperforms System-I in terms of energy efficiency and sustainability. The higher ER, greater EP, lower SE, and improved NEG in System-II highlight the potential of renewable energy in enhancing the energy performance of hydroponic systems while contributing to the sustainability goals of the WEFE nexus.

**Table 7:** Energy indices for producing 1 m^2^ of lettuce in System-I and System-II.

Input	Abbreviation	System-I	System-II
Energy ratio	ER	0.05	0.11
Energy productivity	EP (kg/MJ)	0.07	0.16
Specific energy	SE (MJ/kg)	14.89	6.14
Net energy gain	NEG	− 161.53	− 61.93

### Analysis of CO_2_ emissions for System-I and System-II

The analysis of CO_2_ emissions between System-I and System-II is illustrated in Table 8. The comparison of CO_2_ emissions between System-I and System-II reveals significant environmental advantages associated with the use of PV solar energy in hydroponic systems. System-I produced total emissions of 1.5386 kgCO^2^eq/m^2^, substantially higher than System-II’s 0.0861 kgCO^2^eq/m^2^. The primary driver of emissions in System-I is the electricity used for the NFT pump, which accounts for 94.4% of its total emissions (1.45 kgCO_2_eq/m^2^), that is allies with some studies which have shown that PV solar energy can lower carbon footprints in controlled agricultural environments by replacing fossil fuel-based electricity sources with renewable alternatives^18,27^.

**Table 8:** CO_2_ emissions for producing 1 m^2^ of lettuce in System-I and System-II.

	CO_2_ emissions (kgCO_2_eq/m^2^)		Share (%)	
	System-I	System-II	System-I	System-II
Electricity for the treatment pump unit	0.0077	0.007	0.5	9.00
Steel	0.0083	0.008	0.5	9.7
PE	0.0024	0.0024	0.1	2.8
PVC	0.040	0.040	2.6	46.9
Phosphorous	0.0023	0.0023	0.1	2.7
Potassium	0.0068	0.0068	0.4	7.9
Nitrogen	0.017	0.017	1.1	20.8
Electricity/PV for NFT pump	1.45	0	94.4	0
Total	1.5438	0.0860	100	100

In contrast, System-II eliminates this source of emissions by relying on PV solar energy, highlighting the significant environmental benefit of renewable energy integration. Material-related emissions, such as those from steel and PVC, are comparable between the two systems but take on a larger share of System-II’s emissions profile due to its lower overall emissions. For instance, PVC contributes 46.9% of System-II’s emissions and 2.6% of System-I’s emissions. Nutrient inputs, including nitrogen, phosphorus, and potassium, contribute similarly to emissions in both systems, with nitrogen being the largest contributor (1.1% for System-I and 20.8% for System-II). These findings demonstrate that while System-II achieves a drastic reduction in total emissions by eliminating reliance on conventional electricity, emissions from materials and nutrients remain areas for further optimization. The integration of PV solar energy in hydroponic systems proves to be a transformative step toward sustainability, significantly lowering carbon emissions and aligning with the objectives of the WEFE nexus.

**Fig. 8:**
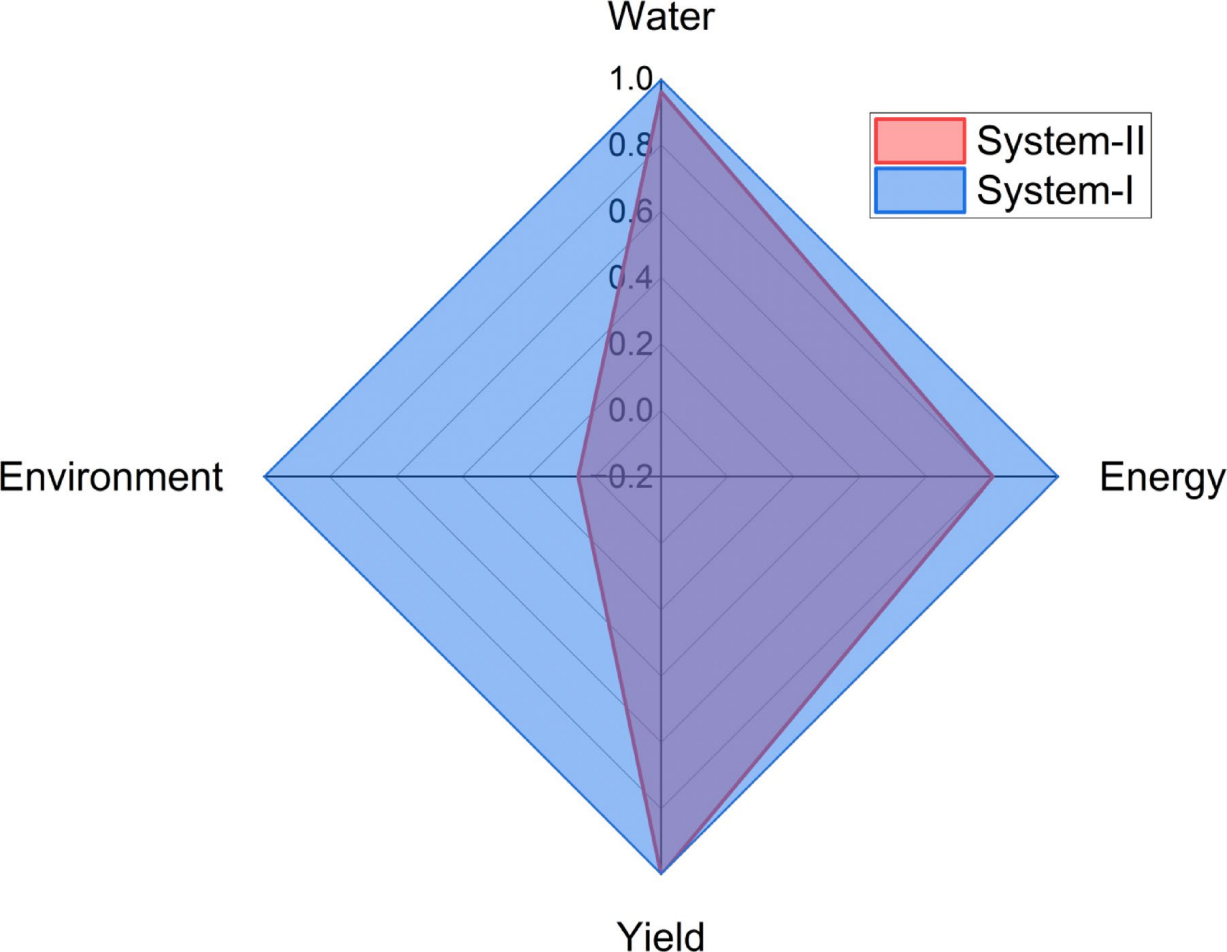
WEFE nexus comparison between System-I and System-II.

Figure 8 provides a comparative analysis of System-I and System-II across the key components of the WEFE nexus: water, energy, food (yield), and environment. Both systems exhibit similar performance in terms of water use efficiency and yield, as indicated by their normalized values reaching the maximum scale on these axes. However, notable differences emerge in energy and environmental performance. System-II demonstrates superior energy efficiency compared to System-I, indicating a more effective use of energy resources. Furthermore, System-II significantly outperforms System-I in environmental impact, suggesting that it offers a more sustainable option with lower environmental burdens. Despite System-I maintaining competitive water and yield outputs, its lower performance in energy efficiency and environmental criteria highlights areas that require further optimization. Overall, the analysis suggests that System-II presents a more balanced and sustainable approach within the WEFE framework, achieving productivity without compromising energy efficiency or environmental integrity.

Table 9 provides a brief comparison of our research work with similar research. A review of related studies reveals that while several have addressed individual aspects of sustainable hydroponic systems, such as the use of smart technologies, renewable energy, or treated water, few have taken an integrated approach. For instance, Saraswathi et al.^57^ and Mishra et al.^58^ focused primarily on smart hydroponic systems, whereas Khang et al.^16^ and Ahmed and Khalid^17^ incorporated renewable energy sources into their designs. Some studies, such as those by Eregno et al.^59^ examined the reuse of treated water. However, Neranjan et al.^18^ considered CO_2_ emissions, and energy efficiency indices were not comprehensively assessed in any of the reviewed works. Furthermore, plant quality was evaluated in only a limited number of cases^26^. In contrast, our study uniquely integrates all these dimensions by combining smart hydroponic farming with PV-powered systems, treated greywater reuse, detailed estimations of CO_2_ emissions, analysis of energy efficiency indices, and evaluation of crop quality. This holistic framework represents a novel contribution to the literature, addressing multiple sustainability challenges concurrently and offering a replicable model for resource-constrained agricultural environments.

**Table 9:** Comparison of our work with similar research.

References	Employs smart hydroponic farming	Uses renewable energy	Uses treated water	Estimates CO_2_ emissions	Estimates energy efficiency indices	Evaluates plant quality
Kappel et al. ^26^	x	x	x	x	x	✓
Saraswathi et al.^57^	✓	x	x	x	x	x
Eregno et al.^59^	x	x	✓	x	x	x
Mishra et al.^58^	✓	x	x	x	x	x
Joshitha et al.^60^	x	x	x	x	x	x
Neranjan et al.^18^	✓	✓	x	✓	x	x
Khang et al.^16^	✓	✓	x	x	x	x
Ahmed and Khalid ^17^	✓	✓	x	x	x	x
Our study	✓	✓	✓	✓	✓	✓

Solar-powered, greywater-fed hydroponic systems hold significant promise for smallholder farmers, urban agricultural initiatives, off-grid communities, and regions facing water and energy scarcity. These systems are especially beneficial in arid and semi-arid areas where conventional agriculture is limited by resource constraints. Their adoption can enhance food security, reduce reliance on freshwater and fossil fuels, and support sustainable urban farming. Optimal performance of such systems is influenced by several key parameters, including solar irradiance, ambient temperature, photovoltaic panel orientation, greywater composition, crop type, nutrient concentration, and the timing of irrigation cycles. Efficient energy storage and system automation further improve reliability and productivity. Understanding and optimizing these parameters are critical to tailoring hydroponic solutions to specific local contexts and maximizing their sustainability and efficiency.

## Conclusions

This study presents a comparative analysis of the performance, energy efficiency, and environmental sustainability of hydroponic systems powered by PV solar energy versus those utilizing conventional electricity, with treated graywater serving as the primary water source. The findings highlight the significant advantages of integrating PV solar energy into hydroponic systems. System-II, powered by PV solar energy, demonstrated superior energy efficiency with higher energy ratios, greater energy productivity, and reduced specific energy consumption compared to System-I, which relied on conventional electricity. Furthermore, System-II achieved a drastic reduction in total CO_2_ emissions, primarily due to the elimination of emissions from electricity use for the NFT pump. The comparative analysis of System-I and System-II reveals similar lettuce yields 11.38 kg/m^2^ per season, with System-I using 161 L/m^2^ and System-II 155 L/m^2^. This results in marginally different WUE values: 0.071 kg/L for System-I and 0.073 kg/L for System-II. For energy consumption, System-II consumes 6.9 kWh/m^2^ per season, compared to 8.6 kWh/m^2^ for System-I, System-II achieves a higher energy productivity (EP) of 1.646 kg/kWh, compared to 1.316 kg/kWh for System-II. In conclusion, the integration of PV solar energy and greywater treated in hydroponic systems represents a sustainable and efficient approach to modern agriculture. By further optimizing material use and nutrient management, these systems can contribute to resilient, low-carbon food production, addressing global resource challenges and advancing the WEFE nexus goals.

Future studies should explore climate-responsive modeling of solar-powered hydroponics under diverse conditions, assess the long-term effects of greywater quality on crop safety, and investigate hybrid renewable energy integration for improved reliability. Additionally, conducting techno-economic and life cycle assessments will help evaluate the sustainability and financial feasibility of such systems compared to conventional approaches. Notably, material-related emissions—particularly from PVC and steel—contribute significantly to the system’s total emissions. Future work should consider incorporating life cycle sustainability perspectives and evaluating more environmentally friendly material alternatives to minimize environmental impact.

## Data Availability

Data available on request from the first author mahmoudabdelhamid@agr.asu.edu.eg.
